# Shear Strength of Fiber Reinforced Recycled Aggregate Concrete

**DOI:** 10.3390/ma13184183

**Published:** 2020-09-20

**Authors:** Mohamed Ghoneim, Ayatollah Yehia, Sherif Yehia, Wael Abuzaid

**Affiliations:** 1 Civil Engineering Department, American University of Sharjah (AUS), Sharjah P.O. Box 26666, UAE; b00039215@aus.edu; 2 Department of Engineering Systems and Environment, University of Virginia, Charlottesville, VA 22903, USA; ay8tb@virginia.edu; 3 Mechanical Engineering Department, American University of Sharjah (AUS), Sharjah P.O. Box 26666, UAE; wabuzaid@aus.edu

**Keywords:** concrete shear strength, recycled aggregate, steel fiber, synthetic and hybrid fibers

## Abstract

In this paper, shear strength of fiber reinforced recycled concrete was investigated. A Self Consolidated Concrete (SCC) matrix with 100% coarse recycled aggregate and different types of fibers were used in the study. Steel (3D and 5D), synthetic and hybrid fibers with a volume fraction of 0.75% were added to the concrete matrix to prepare eight beams. In addition, two beams were cast without fibers as control specimens. All beams were prepared without shear reinforcement and were tested to evaluate concrete contribution to the shear capacity. In addition, optical images were captured to allow for full-field displacement measurements using Digital Image Correlation (DIC). The results showed about 23.44–64.48% improvement in the average concrete shear capacity for fiber-reinforced beams when compared to that of the control specimens. The percentage improvement was affected by fiber type and the steel fiber beams achieved the best performance. The addition of the fiber delayed the crack initiation and improved the post-cracking and ductile behavior of all beams. Moreover, the experimental results were compared to those predicted by codes and proposed equations found in the literature for concrete strength with and without fibers.

## 1. Introduction

Introducing recycled concrete aggregate (RCA) to the construction industry is an effort to preserve natural resources and to achieve sustainability [1,2,3,4,5,6]. However, concerns about variability of recycled aggregate (RA) properties have limited the use of RCA in structural applications. Several research efforts were devoted to evaluating fresh and hardened concrete properties containing different percentages of coarse recycled aggregate [3,4,5,6,7,8,9,10,11,12,13,14]. However, research conducted on the structural behavior of RCA is limited and often contradictory. In general, flexural and shear crack patterns of 100% replacement of recycled aggregate concrete and conventional normal weight concrete are identical; nonetheless, shear capacity in recycled aggregate concrete is relatively less than that of conventional concrete [15,16,17,18,19,20,21,22,23,24]. Addition of fibers and supplementary cementitious materials was an approach to improve the mechanical properties and flexural strength of recycled aggregate mixes. Moreover, fiber reinforcement can be used to improve the shear capacity of the concrete and will help resist the brittleness shear failure and provide a more ductile behavior including post-cracking tensile strength [25,26,27,28,29,30,31,32,33,34]. Resistance to shear failure is typically provided by concrete shear strength (*v_c_*) and shear reinforcement (*v_s_*). Stirrups and bent bars are the commonly used shear reinforcement in design. Recently, the American Concrete Institute (ACI-318) standard recognized fiber reinforcement as shear reinforcement if the fiber volume fraction is ≥0.75% [35]. Other factors such as the un-cracked compressive concrete zone, aggregate interlock, dowel action, longitudinal and shear reinforcement, span-to-depth ratio, and compressive strength affect the shear capacity and contribute to the overall shear resistance [15,16,17,35,36,37,38,39,40,41,42,43,44,45,46].

### 1.1. Concrete Contribution to Shear Resistance

Shear strength provided by concrete is usually presented as a function of concrete compressive strength (*fc′*). However, this value is affected by aggregate strength; a recent study by Yehia et al. [14] showed that aggregate type plays a major role in concrete compressive strength. Furthermore, codes of practice recognize the effect of different concrete types by introducing a modification factor to account for the aggregate type, especially when it comes to shear and bond calculations. For example, ACI 318 [35] uses λ value to account for lightweight aggregate in shear and bond equations. Aggregate shape and surface texture influence aggregate interlock which is one of the main contributors to concrete shear resistance. Aggregate physical properties and bond with cement paste are also valid when it comes to recycled aggregate (RA) because of the variability of the aggregate quality and particle distribution. However, there is an argument that RA has a rough surface due to the crushing process, which should lead to an improvement in concrete shear resistance. On the other hand, mortar adhered to the aggregate particles increases; porosity, and absorption capacity create a weak interfacial transition zone (ITZ) that might lead to weak aggregate interlock and reduced shear resistance. Both arguments are valid and thus, to overcome some of these concerns, a careful mix proportioning and mixing procedure is required. These procedures can benefit from the surface roughness to improve concrete contribution to shear resistance.

### 1.2. Shear Strength of RCA

Table 1 [15,20,21,22,47,48,49,50,51,52,53,54,55,56,57,58,59,60,61,62,63,64,65,66] highlights the literature focusing on RCA’s effects on shear strength, with the last three rows focusing specifically on the effect of fibers addition on shear strength of RCA. RCA generally decreases shear strength in beams as well as cylindrical push-off specimens [22,50,52,55,56,57,58,61], in addition, other research efforts indicated that shear strength is decreasing as the RCA replacement ratio increases [15,63]. This reduction is caused by a myriad of factors, such as remaining mortar on RA and higher water absorption due to increased porosity in RA in comparison to normal weight aggregate (NWA) [33]. RA has a weaker adhesion performance in the ITZ [67] when compared to NWA. This causes micro-cracks to form in the ITZ, which lead to lower shear strength of RCA [1]. In addition, RCA also contains multiple transition zones, an ITZ between fresh aggregate and the pre-existing mortar on RA and an ITZ between the pre-existing mortar and fresh mortar [1,67]. The ITZ depend on aggregate type and water transport between cement paste-aggregate interface during hydration [1,9,10,68]. Therefore, the weak adhesion performance, along with the greater ITZs, have an adverse effect on the mechanical properties of RCA [9,67]. A proportioning method can improve the shear strength of conventional beams with RCA, as proposed by [48]. Treated RCA in beams provides higher shear strength than untreated RCA [51,59]. When RCA replaces a smaller grade of coarse aggregate, there is no reduction in shear strength, provided RCA replacement is less than 16% [56]. Beams with shear reinforcement and RCA had minor differences in shear strength with various levels of RCA replacement [20,54]. The consensus is when experimental RCA results are compared with existing models, shear strength is conservative. However, when the RCA replacement is greater than 50%, this no longer has merit [22]. Existing models are unable to predict results for specimens with RCA and shear reinforcement [57].

Utilizing steel fiber to improve shear capacity was investigated in the early 90′s [69,70,71]. Steel fibers control the spreading of cracks in a specimen, and thus reduce the width of cracks [65]. Research efforts by [69,70,72] focus on the improvement of shear strength when steel fibers are added, and in addition, models/equations capable of predicting shear contribution of fiber reinforced concrete and the improvement of shear strength are proposed by [70,73]. When short steel fibers are added in RCA specimens, the reduction in shear strength is negated, and additional shear strength is provided [64], which could be attributed to the fiber ability to bridge cracks and improve the ITZ performance in RCA [67]. Increasing the RA replacement ratio improved the shear behavior with different transverse reinforcement spacing in fiber reinforced concrete [65], and shear strength of beams considerably improves in beams, cylinders, cubes, and prisms when fibers are added to specimens with RCA [66]. The available literature, to the authors’ knowledge, that discusses the ideas presented in the current investigation to evaluate the contribution of different types of fibers to shear strength of RA is limited, highlighting the need for more research efforts to encourage the use of RCA in structural applications.

### 1.3. Code Equations to Calculate Concrete Contribution to Shear Strength

Common code equations to calculate concrete contribution to shear strength are summarized in Table 2. Some of these equations are used for concrete with normal weight aggregate; however, many studies for RAC adopted them to evaluate their applicability for recycled aggregate concrete.

Recently, ACI318-19 [35] adopted a new equation to be used in the case of transverse reinforcement if *A_v_* is less than the minimum required transverse reinforcement A_v,min_. In addition, both ACI 318-19 equations are recommended for normal weight aggregate and can be used for lightweight aggregate concrete with a modification factor *λ*, to reflect the difference in properties of lightweight concrete when compared to normal weight concrete of the same compressive strength. Applicability of the Equations in Table 2 to concrete with 100% recycled aggregate is discussed in the current study.

In this study, a high-strength self-consolidated concrete (SCC) matrix with 100% coarse recycled aggregate and different types/configurations of fibers were used to assess fiber contribution to shear resistance. Tests were conducted to evaluate concrete contribution to shear resistance. In addition, optical images were captured to allow for full-field displacement measurements using Digital Image Correlation (DIC). The results were compared to those calculated from different codes and proposed equations found in the literature.

### 1.4. Research Significance

There are many efforts to achieve sustainability in the construction industry by encouraging the use of recycled aggregate; however, utilizing recycled aggregate in structural applications is limited because of the contradicting results about shear performance of the recycled concrete aggregate (RCA). Fiber addition to improve the mechanical properties of the RCA could provide a solution to increase the demand on recycled aggregate. This investigation will play a role in sustainability efforts and, hence, contribute to the preservation of natural resources.

## 2. Experimental Program

The main objective of the experimental program is to evaluate the effectiveness of fiber addition for the purpose of improving shear resistance of recycled aggregate concrete. Steel (3D and 5D), synthetic and hybrid fibers (mix of steel 5D and synthetic fibers) with a volume fraction of 0.75% were added to the concrete matrix to prepare eight beams. In addition, two beams were prepared without fibers as control specimens. Tests were conducted according to the American Society for Testing and Materials (ASTM) specifications and British Standards (BS). Results of the mechanical properties and shear tests were compared to those found in the literature and codes of practice.

### 2.1. Materials

#### 2.1.1. Recycled Aggregate

**Figure 1:**
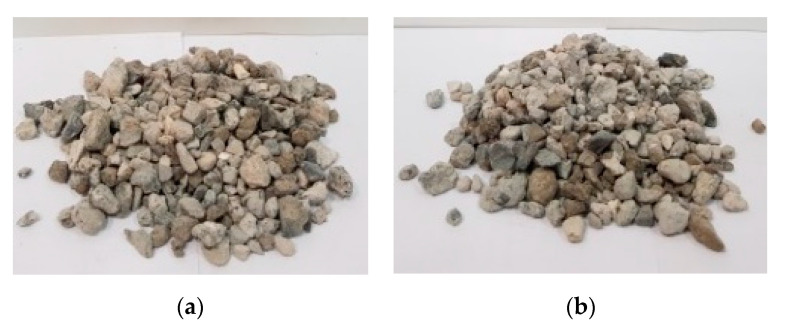

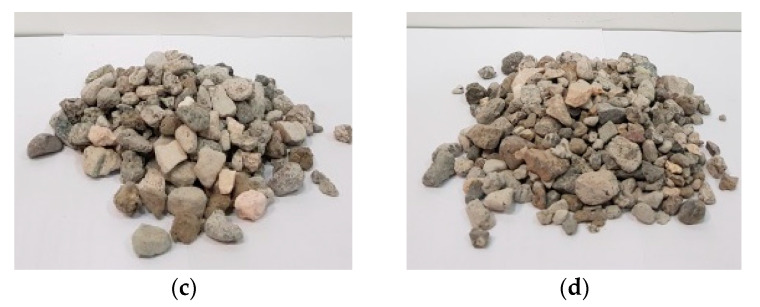
Different batches of recycled aggregate particles. (**a**) Batch 1 (P1), (**b**) Batch 2 (P2), (**c**) Batch 3 (P3), (**d**) Batch 4 (P4).

Recycled coarse aggregate used in this research was delivered from a local recycling plant in Sharjah, UAE. Samples from four different batches were collected to evaluate the physical and mechanical properties of the aggregate. The main objectives of the aggregate evaluation are to check the variability in the properties among the four batches and to ensure availability of aggregates with similar properties during the investigation. The samples were labeled P1, P2, P3, and P4, as shown in Figure 1. Physical properties such as absorption capacity and specific gravity for the four batches were evaluated to consider their effect during the mix proportioning and mixing. In addition, Los Angeles abrasion test was performed to provide indication of aggregate strength. Tests were conducted according to the American Society for Testing and Materials (ASTM) specifications [74,75,76,77]. Table 3 summarizes the results of the physical properties of the recycled aggregate.

The test results indicated that the aggregate had a high absorption capacity, low specific gravity, and high weight loss (weak aggregate). This could be attributed to the high porosity characteristics of the recycled aggregate and the old mortar adhered to the original coarse aggregate.

**Figure 2:**
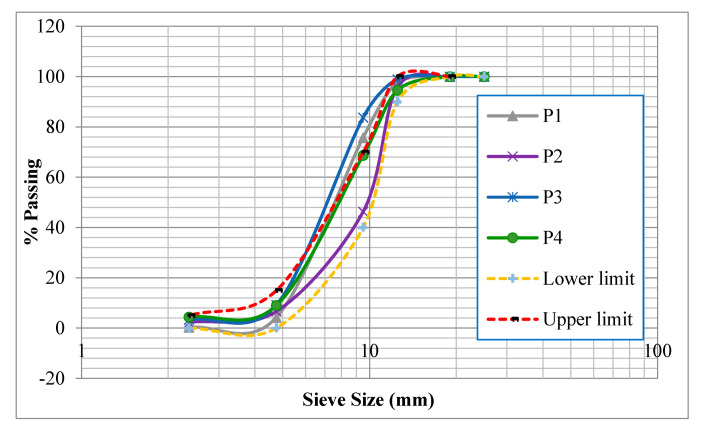
Sieve analysis of the four batches.

In addition, sieve analysis according to ASTM C33/C33M [77] was done to determine the gradation and particle distribution of the four batches, as shown in Figure 2. In addition, the upper and lower limits for the aggregate size (4 to 14 mm) are also shown in Figure 2. Particle distribution for batch P2 was different from those of the other batches; therefore, batch P2 was excluded from the investigation.

#### 2.1.2. Fibers

**Figure 3:**
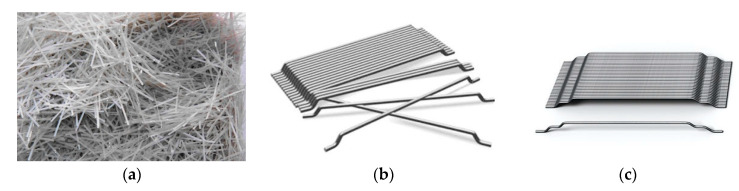
Fibers used in the investigation. (**a**) Strux(90/40) Synthetic, (**b**) 3D Steel Fiber, (**c**) 5D Steel Fiber.

Four different combinations of fibers were considered in the current study. Two configurations of steel fibers (3D and 5D), synthetic and hybrid (a blend of 5D and synthetic) fibers, Figure 3. The main difference between 3D and 5D steel fibers is the configuration at both ends. For the 3D fiber, two bends at each tip of the utilized steel wire results in three surfaces. Such a configuration improves the anchorage properties. The 5D steel fibers have extra bends resulting in additional surfaces (i.e., five) for improved anchorage strength and pull-put capacity. Table 4 summarizes properties of all fiber types provided by the manufacturer [78,79]. A volume fraction of 0.75% of all fiber types (3D, 5D, and synthetic) was used to prepare four concrete batches. In the case of hybrid fibers, the percentage was divided equally for both fiber types.

#### 2.1.3. Other Materials

Portland cement Type I (specific gravity [SG] = 3.14) and silica fume (SG = 2.22) were the cementitious materials considered in the investigation. Normal weight dune sand (particle size 100% passing 0.6 mm, SG = 2.60) and coarse sand (maximum particle size 4.75 mm, SG = 2.60) were used in the current evaluation as fine aggregate.

### 2.2. Mix Proportioning and Mixing Procedure

Five self-consolidated concrete mixes were prepared in this study. One mix without fiber, control mix, and four mixes with fibers. The mixes were labeled as RCA-fiber type, for example, RCA-3D refers to recycled concrete-steel fiber 3D. All mixes prepared in the lab were proportioned using the absolute volume method, summarized in Table 5. Volume fractions of the cementitious materials, recycled coarse aggregate and *w*/*c* ratio were the same for all mixes. GLENIUM SKY 502 superplasticizer, BASF Construction Chemicals, Dubai, UAE, was used in all mixtures to achieve the desired flowability. The recommended dosage of the superplasticizer is 0.6 to 1.5 L per 100 kg of total cementitious material. The target strength was 60 MPa and the proportioning was based on a normal weight self-consolidated concrete mix [80] with a target strength of 70 MPa.

The absorption capacity of the recycled coarse aggregate was about 6%, which is higher than that of normal weight aggregate (typically ~1%). Therefore, a mixing process proposed by Yehia et al. [68,80] for pores aggregate with high absorption capacity was followed for all mixes with RCA. The recycled aggregates were pre-wet 30 min prior to mixing with part of the mixing water (about 6% of the RCA aggregate weight); in addition, about 6% of the cement and silica fume weight were added during the pre-wet process. This process helped improve the workability during mixing and enhanced the bond strength between the aggregate and the cement paste [68,80].

### 2.3. Testing Program

The testing program consisted of two parts: the first part focused on the evaluation of the mechanical properties of all concrete mixes included in the study. The second part focused on the evaluation of the shear capacity of concrete using structural beam testing. Compressive strength, splitting tensile strength and flexural strength were used to evaluate the mechanical properties according to British Standards (BS) [81] and ASTM [82,83]. Table 6 summarizes the tests, number of samples, sample size, age at testing, and specifications followed during testing.

#### 2.3.1. Sample Preparation

**Figure 4:**
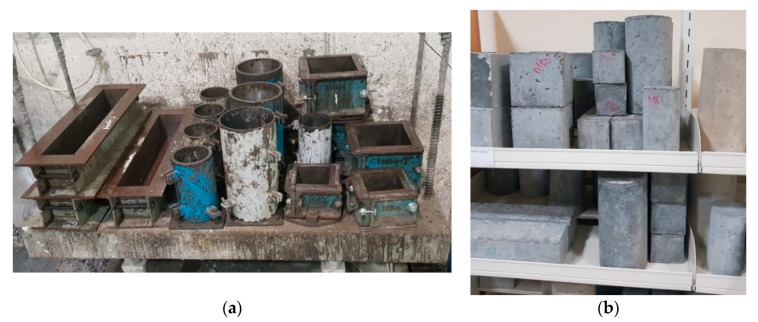
Samples preparation for mechanical properties. (**a**) Molds used for sample preparation, (**b**) Samples for mechanical evaluation.

Cubes, cylinders, and beams were prepared from the same mixes used to prepare the beams for the shear tests. Figure 4 shows the molds used and samples after casting. All samples were cured for 7 days using wet burlap and then, were kept in the laboratory until the testing date.

**Figure 5:**
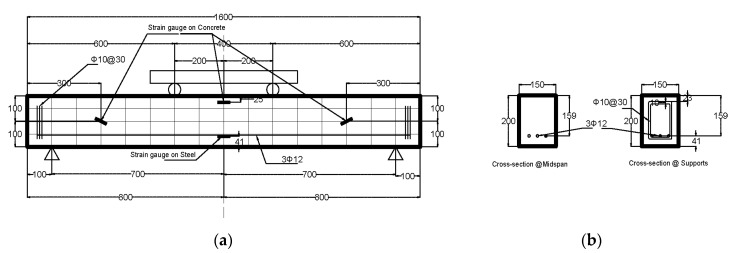
Beam and cross section details. (**a**) Elevation reinforcement and strain gauges location, (**b**) Beam cross-section. All dimension are in mm.

Ten beams, two from each concrete mix, with a rectangular cross section of 150 mm width and 200 mm depth and 1600 mm long were prepared to evaluate the shear capacity of concrete for different mixes, as shown in Figure 5. Three 12 mm diameter bars and two 12 mm bars were used as bottom reinforcement and top reinforcement, respectively, with all bars having a length of 1540 mm. The percentage of the longitudinal reinforcement ρ (A_s_/bd) = 1.4%, which was selected to ensure that the beams fail in shear. No shear reinforcements were used along the beam; however, three bars of 8 mm closed stirrups were used at the support locations to avoid stress concentration during testing, to secure the lifting hooks and prevent failure during setup.

**Figure 6:**
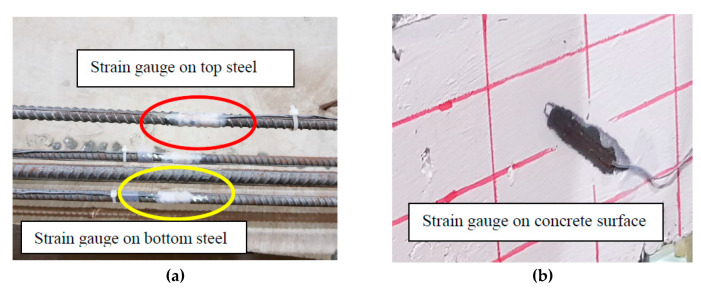
Strain gauges on steel bars and concrete surface. (**a**) Strain gauges on top and bottom steel bars, (**b**) Strain gauges on concrete surface.

**Figure 7:**
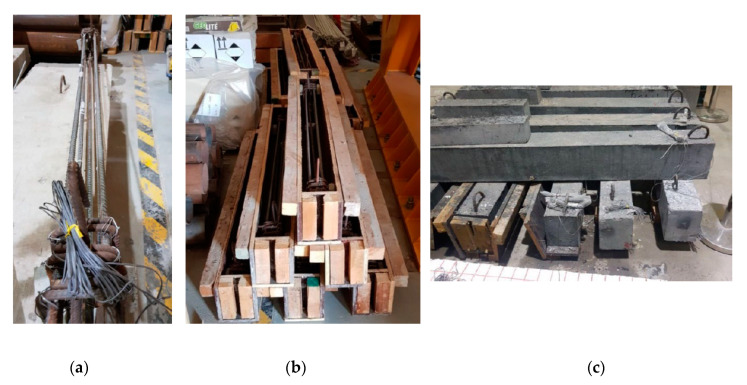
Samples during preparation. (**a**) Steel reinforcement with strain gauges, (**b**) Formwork before casting, (**c**) Samples after curing.

Three strain gauges, Tokyo Measuring Instruments Laboratory, Tokyo, Japan, were placed on the steel bars, two on the bottom bars, and one on the top bar, as shown in Figure 6. In addition, three strain gauges are installed on the concrete surface before testing, two of which were placed at 45 degrees to the beam axis and perpendicular to the expected shear failure location in the shear span. The third gauge was placed on the compression zone. Figure 6 and Figure 7 show the strain gauges on the steel bars, on the concrete surface, and part of the beams during preparation and after casting.

#### 2.3.2. Test Setup

**Figure 8:**
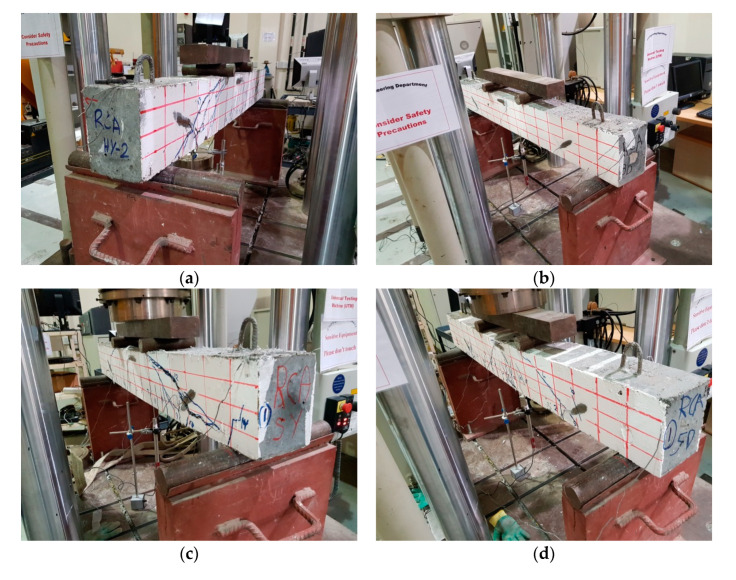
Test setup—beams under loading. (**a**) RCA-HY_1, (**b**) RCA-3D-1, (**c**) RCA-5D-1, (**d**) RCA-SY-1.

A four-point loading test setup, Dubai, UAE, was used to test all beams utilizing an Instron servo-hydraulic load frame, MA, USA, with a displacement control and loading rate of 0.6 mm/min. The supports were placed at 100 mm from both ends of the beam. The load was applied on a spreader beam to have two point loads with a distance of 500 mm from each support, shown in Figure 5. This arrangement provided a shear span-to-depth ratio (a/d) of 3.14, which is greater than the recommended by the code (a/d > 2) [35]. Figure 8 shows samples from different beams during testing.

**Figure 9:**
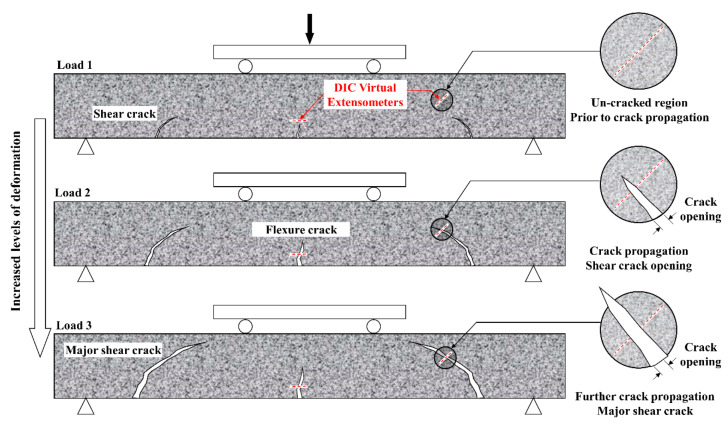
Schematic explaining the use of virtual extensometers for crack opening measurements.

To enable full-field strain measurements using DIC, a speckle pattern consisting of a white background and black speckles was added to the back surface of each beam. Optical images of the monitored region were collected during loading at a rate of 1 image every 2 s, to failure. All correlations were conducted using a commercial DIC software (Vic-2D 6 from Correlated Solutions, Irmo, SC, USA). Virtual extensometers (VE) were utilized to provide quantitative information about crack openings throughout the loading history. As shown schematically in Figure 9, measurements were made at several locations along the length of major developed cracks. Initially, and prior to crack initiation, the added extensometers exhibit insignificant deformation. However, once a crack initiates and propagates through the monitored region (i.e., passes through the VE), a clear increase in opening values is detected. The opening level increase as the crack propagates reaching significantly larger magnitudes close to failure.

## 3. Results

### 3.1. Evaluation of Mechanical Properties

#### 3.1.1. Compressive Strength and Splitting Tensile Strength

The main testing event for the mechanical properties and shear test was scheduled after 90 days of mixing to ensure complete hydration, which is recommended for concrete prepared with porous aggregate. Table 7 summarizes the results for the compressive strength and splitting tensile strength for all mixes and percentage difference compared to those of the control mix. The recycled aggregate concrete mixes with steel fibers (3D and 5D) and the hybrid mix (5D steel fiber and polypropylene fiber) had an increase in compressive strength by 5.4%, 17.3% and 9.3%, respectively compared to that of the RCA. However, recycled aggregate concrete with polypropylene fiber had a slight reduction in compressive strength with respect to the RCA of about 0.78%.

RCA with 3D, 5D, and polypropylene had an increase in the split strength of 72.5%, 123.47%, and 93.5%, respectively. Moreover, for RCA-HY the increase reached up to 140.8% compared to that of plain RCA. Table 8 illustrates different failure modes for cubes and cylinders from all mixes. RCA cubes and cylinders tested for compressive and splitting tensile strengths, respectively, showed typical failure modes. However, the energy absorption capacity of the fibers in the RCA-3D, RCA-5D, RCA-SY, RCA-HY helped controlling the cracks and non-explosive failure modes for both compressive and splitting strengths samples were observed. Some splitting tensile strength samples were broken to examine the cement-aggregate bond and fiber distribution.

#### 3.1.2. Flexural Strength

**Figure 10:**
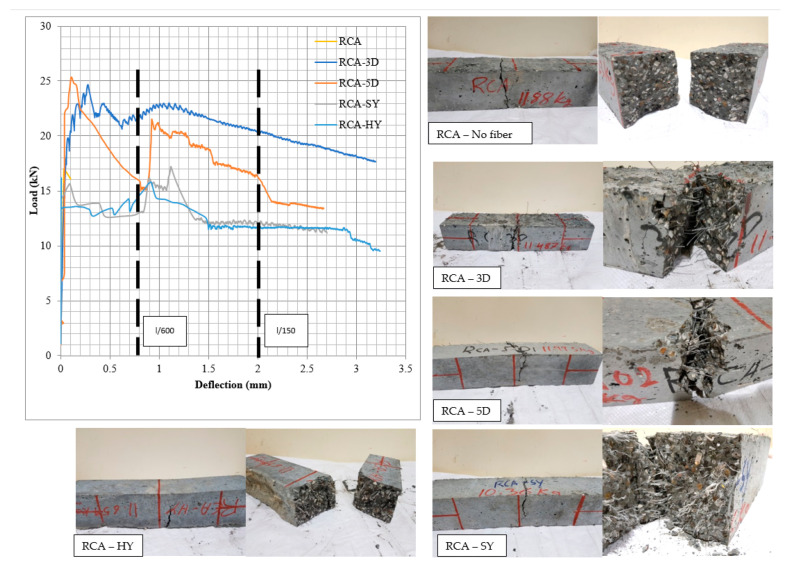
Load-deflection curves for flexural performance of fiber-reinforced recycled concrete and mode of failures.

Table 9 and Figure 10 summarize the average results of the 100 mm × 100 mm × 500 mm prisms under static flexural loading in accordance with ASTM C1609/C1609M [83]. This test provides the first peak, peak and residual loads with their corresponding stresses, toughness and the flexural strength ratio. RCA showed a typical brittle failure, whereas, samples with fibers provided residual capacity after reaching the peak stress. The residual strength, which characterize the prisms residual capacity of mix after cracking, is calculated at specified deflections, L/150 and L/600 of the span length. Furthermore, the peak flexural strength increased by 23.68% and 74.24% for steel fibers 3D and 5D, respectively; however, there was an increase of 11.04% and 8.32% for both synthetic and hybrid mixes, results which reflect the effect of fiber type and configuration. Typical failure mode for fiber-reinforced prisms showed improved deflection and post cracking behavior. In addition, all prisms were broken to examine the fiber distribution and cement-aggregate bond.

### 3.2. Concrete Contribution to Shear Resistance

**Figure 11:**
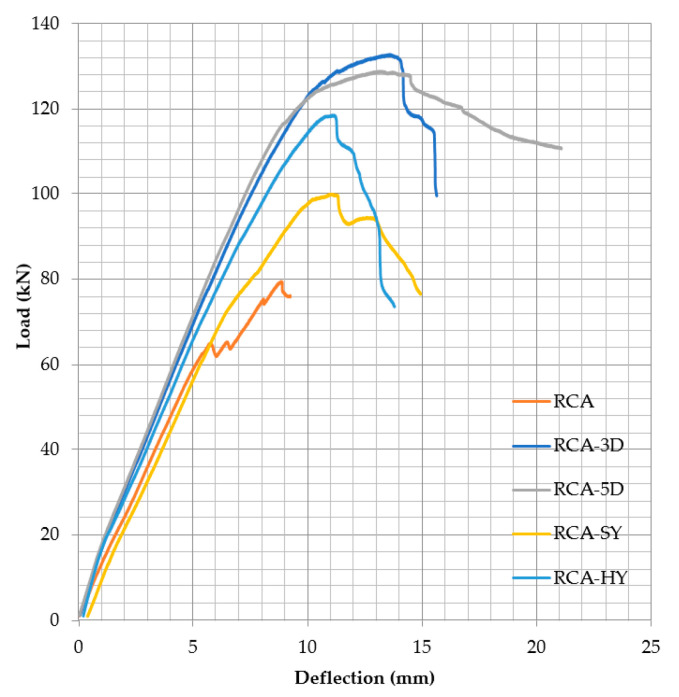
Load-deflection—average results for beams from each mix.

Two beams from each mix were subjected to 4-point load evaluation. The main goal is to determine the concrete contribution to shear resistance (*v_c_*). Therefore, no shear reinforcement (stirrups) was used and the shear span-to-depth ratio (a/d) of 3.14 was maintained during the testing as discussed in Section 2.3.1. The average load-deflection curves for the control and fiber reinforced recycled concrete beams are shown in Figure 11. The ultimate load, failure load, shear load, ultimate deflection, stiffness, and failure modes for the ten beams are summarized in Table 10. In addition, the shear loads were compared to that of the control samples. The control samples without fibers achieved shear resistance (*v_c_*) greater than that predicted by the current ACI 318 equation [35] for normal weight aggregate indicated by the α > 0.17 if A_v_ ≥ A_v,min_ or α′ > 0.66 if A_v_ < A_v,min_. In addition, the fiber addition clearly improved the concrete shear resistance and overall performance of all beams. The improvement was influenced by the fiber type and configuration. The α and α′ are affected by the fiber type and aspect ratio since the same fiber volumetric ratio was used for all types.

### 3.3. Full-Field Deformation Measurments—Crack Opening

**Figure 12:**
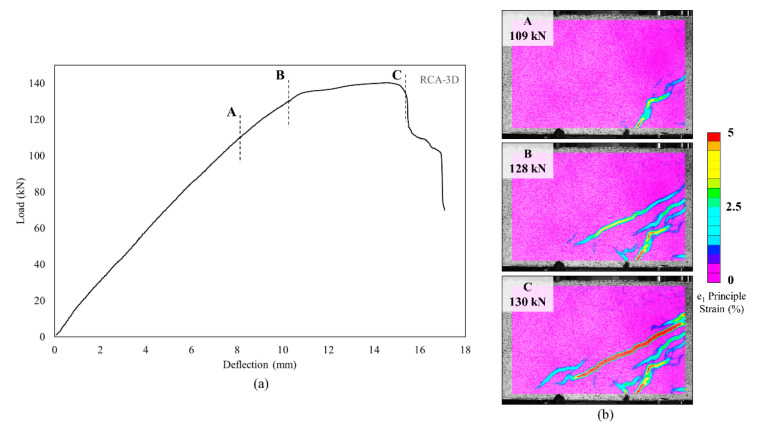
(**a**) A representative load-deflection curve. (**b**) Strain contour plots at different loading levels (A–C).

The load vs. mid-span deflection for a representative beam, RCA with 3D fiber reinforcements, is shown in Figure 12a. Strain contour plots at different loading levels are shown in Figure 12b. The clear strain localization band observed in A at a load of 109 kN is associated with the development of a shear crack. Significant and pronounced propagation of the observed crack is detected at higher loads (see states B and C at 128 and 130 kN, respectively). The formation of an additional crack was also detected as shown in the strain contour plots at B and C.

**Figure 13:**
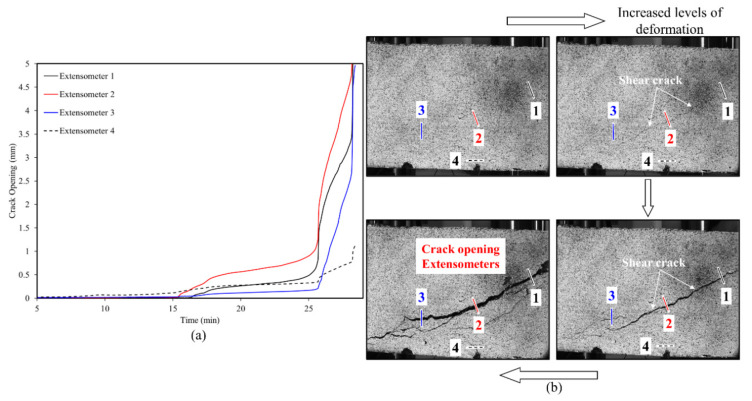
(**a**) Crack width versus time for the RCA-3D case shown in Figure 12. Results from 4 different VE are shown. (**b**) Optical images captured at different deformation levels. The locations of the 4 VE across the length of the crack are marked.

As explained above, virtual extensometers (VE) were added along the path of the major cracks to monitor the crack opening levels. The evolution of crack width with time (i.e., continued loading) is shown in Figure 13a for 4 different VEs. Initially, and prior to crack initiation, the measured crack openings for all locations are small. Notable inflections (i.e., clear increase in crack opening) were observed for all monitored VE. The time, or load, at which this transition takes place, marks the onset at which the crack reached the VE location. Subsequent loading results in further increase in the measured crack opening. The sharp and rapid amplification seen at around 25 min of loading highlights the deformation and development of major cracking close to failure. Similar trends are clear from VE 1–3, which were all, added along the length of the major shear crack. Extensometer 4 (shown with a dashed black line) was relatively different compared to all other extensometers. This particular measurement was made across a flexure crack as opposed to the shear crack in VE 1–3.

The maximum crack opening in this case was much lower compared to the shear crack, as expected, since shear failure is dominant.

**Figure 14:**
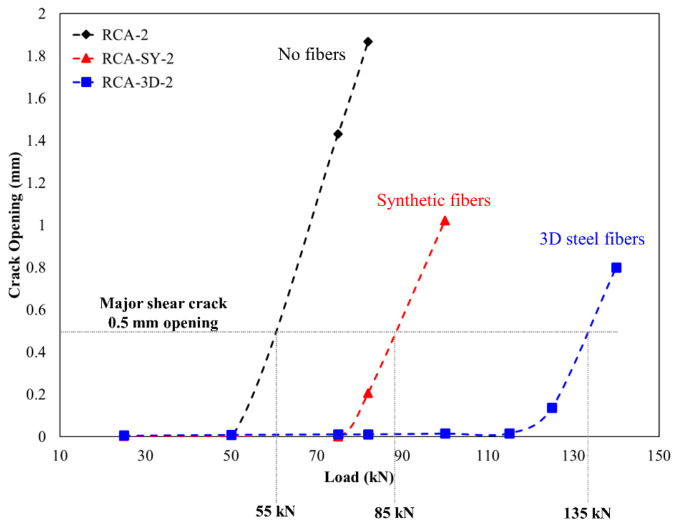
Major shear crack opening versus load for three different cases.

The intrinsic toughening introduced by the use of fibers is expected to affect the cracking and post cracking behavior of concrete beams. The use of VE as shown above enables the quantitative assessment of cracking behavior and can therefore elucidate the effect of different fiber addition. Figure 14 shows the crack opening versus load for three representative beams; the no fiber (only RCA), synthetic fibers, and 3D fibers cases. The data from only one VE is shown for each of the conditions. The extensometers are representative of the major shear crack in each of the considered beams. A clear difference in response is observed. For example, the load at which the crack opening starts to increase, (which is associated with the initiation of cracks) grows with the introduction of fibers, both synthetic and 3D (see the loads axis in Figure 14). The load at which the major shear crack reaches an opening magnitude of 0.5 mm is also shown in Figure 14. With no fiber reinforcement, this cracking level is reached at a load of 55 kN. The introduction of synthetic fibers increases the load at which this magnitude of failure is observed to 85 kN while 3D fibers exhibits a superior response with a significantly higher load of 135 kN (all marked in Figure 14).

## 4. Discussion

All concrete mixes achieved the target compressive strength ≥ 60 MPa. The improved compressive strength and other mechanical properties of concrete with 100% recycled coarse aggregates could be attributed to the mixing process which started by soaking the recycled aggregate before mixing with water and cement/cementitious materials. This process improved the ITZ and enhanced the cement-aggregate bond strength. Detailed discussion about improvement of the ITZ due to the pre-wet/soaking process, addition of silica fume, and fibers/ITZ relation were discussed elsewhere by References [9,68,84,85]. Furthermore, the improvement of the mechanical properties increased the benefits from the addition of fibers, and, hence, improved the concrete shear capacity.

### 4.1. Effect of Fiber Addition on the Mechanical Properties

In general, the main goal from the fiber addition is to improve the tensile and flexural strengths. This was clearly achieved in the current study by improving the splitting tensile strength by 72–140% and the flexural strength by 8–72% by all fiber types compared to that of the control strength. This improvement is attributed to the fibers’ ability to delay crack initiation and improve post-cracking behavior. However, the type of fibers influences the percentage of improvement.

#### Effect of Fiber Type on the Mechanical Properties

Table 7 and Table 9 showed that steel fibers outperformed the synthetic fiber and hybrid fiber in splitting tensile and flexure strengths. The high pullout capacity and stiffness of the steel fibers contributed to this performance. However, the hybrid fibers combined the benefits from both steel and synthetic fibers, which led to high splitting tensile and compressive strengths.

Both types of steel fibers provided similar improvement; nevertheless, fiber configuration provided distinct performance, which was clear by the 5D fibers. The configuration of the 5D fibers provided special anchorage that controlled crack growth, propagation and improved post-cracking behavior. The 5D fiber is the only fiber, in the current study that led to an increase in compressive, splitting tensile, and flexure strengths. It is important to note that the volume fraction of all fibers used in the investigation was 0.75%.

### 4.2. Comparison of Mechanical Properties with Results from Literature

Table 11 summarizes several studies in the literature that discuss the effect on different strengths when steel fibers, recycled steel fibers, and synthetic fibers are added to RCA. Recycled fibers, as well as most synthetic fiber studies, reduce the compressive strength of RCA [32,40]. The studies in the literature differ on how compressive strength is affected when steel fibers are added to RCA, as it may cause an increase or reduction [27,29,30,34,60,66,86]. Tensile and flexural strength are improved when fibers are added to RCA, regardless of the type of fiber [29,30,34,40,60,66,86]. However, other studies have concluded that steel fibers have an inconsiderable effect on mechanical properties in RCA [27,60].

The current study evaluates the effect of 3D and 5D steel fibers, synthetic fibers and a hybrid mix including 5D steel fibers and synthetic fibers on RCA. 3D and 5D steel fibers are still a new phenomenon in regards to including them in RCA and have not been discussed at length in the literature. 3D, 5D, and hybrid fibers have an overall improvement in the mechanical properties of RCA, with synthetic fibers having a nominal decrease on the compressive strength of RCA. As shown in Table 11, 5D steel fibers in RCA provide the highest improvement in compressive and tensile strength in comparison to the provided studies. These fibers also have one of the highest improvements in flexural strength in comparison to the provided studies. The current study illustrates that the different types of fiber improved the mechanical properties of the specimens, with 5D steel fibers providing the largest improvement in compressive, tensile and flexural strength. There are also limited studies available on hybrid steel fibers, when the hybrid fibers are added; it offsets the reduction in compressive strength synthetic fibers have on RCA. Hybrid fibers also increase tensile strength beyond the contribution 5D steel fibers alone provide, and these fibers provide the highest improvement in tensile strength in comparison to the discussed studies. However, hybrid fibers have the lowest improvement in flexural strength when compared to 3D, 5D, and synthetic fibers in the current study.

### 4.3. Effect of Fiber Addition on Shear Performance

The effect of fibers on load versus displacement, ultimate capacity, ductility, stiffness, cracking behavior, and failure mode are discussed in this section. Table 12 shows crack distribution and failure modes for all beams. Seven beams failed in shear; however, three out of four beams reinforced with steel fibers failed in flexure (concrete crushing).

#### 4.3.1. Effect of Fiber on Concrete Shear Capacity

The volume fraction of fibers (0.75%) selected in this study is the minimum recommended by ACI-318 [35] for fibers to contribute to the shear capacity. All fiber reinforced RCA beams achieved high shear capacity compared to that of the control beams. The percentage increase was 64.48%, 59.23%, 47%, and 23.4% for the 3D, 5D, HY, and SY, respectively. The 3D and 5D steel fibers achieved the highest performance—especially the 5D, which improved the flexural strength and contributed to improving the concrete shear resistance. The fiber reinforced RCA beams with 5D failed in flexure, although the longitudinal reinforcement ratio (ρ) was selected to ensure that all beams should fail in shear. The improved anchorage configuration of the 5D controlled the shear cracks and shear failure was not the case for both beams. In this study, the synthetic fibers achieved the least improvement; this could be attributed to the low pullout capacity and lack of anchorage. Meanwhile, the hybrid fibers (5D and synthetic) provided combined benefits from both the steel and synthetic fibers. The percentage increase in the capacity was closer to that of the steel fibers and the deformation was close to that of the synthetic fibers.

**Figure 15:**
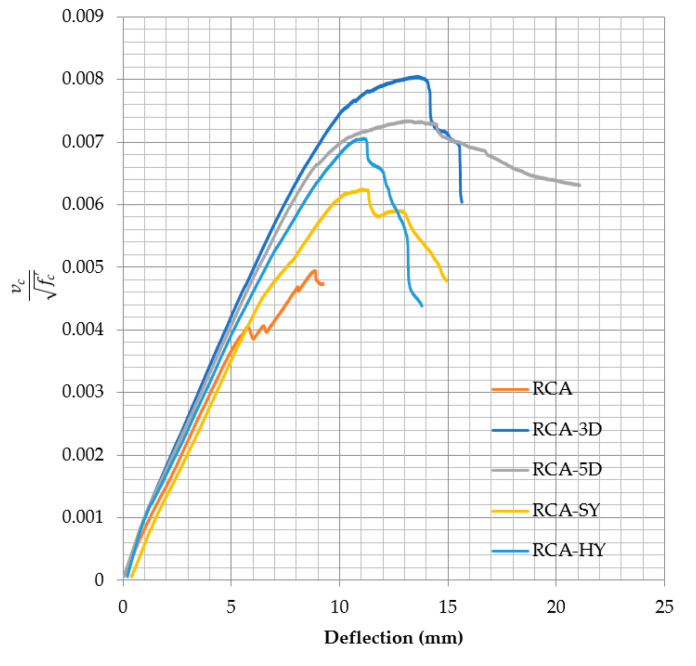
Normalized shear resistance vs Deflection.

To eliminate the effect of the compressive strength, the results were normalized and the shear load was divided by the √(*f_c_’*), as shown in Figure 15. The beams with steel (3D, 5D, or hybrid) fibers showed better performance and high shear capacity compared to that of the control and SY beams. From Figure 15, the 3D beams achieved high shear capacity; however, it is important to remember that the benefits from the 5D fibers were not fully utilized because the beams failed in flexure after achieving the flexural design capacity. The normalized results did not change from the performance since there was no significant variation in the compressive strength of all mixes.

#### 4.3.2. Effect Fiber on Stiffness

Figure 10 shows that the fiber addition resulted in increase of elastic stiffness by 62.97%, 61.55%, 35.56%, and 20.11% for 5D, 3D, HY, and SY beams, respectively, whereas the plastic stiffness increased by 133.92%, 115.9%, 107.25%, and 34.74% for HY, 3D, SY, and 5D, respectively, compared to that of the RCA control beam. The plastic stiffness of the 5D is the first slope of the curve; however, the load-deflection curve for the 5D showed multiple curvatures. Performance of fiber RCA beams based on ductility index were 5D, 3D, HY, and SY. However, based on the maximum deflection the performance was 3D, 5D, HY, and SY. The steel fibers alone or added to the hybrid mix improved both the elastic and plastic stiffness of the beams due to high stiffness and anchorage capabilities of the fibers.

It is important to note that the recommended volume fraction of the synthetic fibers by the manufacturer is 0.5%. Therefore, it was clear that using the same volume fraction (0.75%) of synthetic fibers to maintain the comparison with other fiber types, negatively affected the contribution of synthetic fibers to the overall performance. Due to the difference in the fibers densities, number of synthetic fibers is significantly higher compared to that of steel fibers, which could be attributed to the reduced bond between the fiber and surrounding concrete. Consequently, limiting the improvement in the crack initiation, deflection and capacity.

#### 4.3.3. Effect of Fiber on Crack Initiation, Crack Pattern, and Failure Modes

Initiation of cracks during loading were monitored and propagation were marked on the concrete surface. Crack patterns for all beams are shown in Table 12. In addition, crack patterns, corresponding loads, strain in the bottom steel bars and concrete surface are summarized in Table 13.

An initial flexural crack was observed at the center of all specimens. Other flexural cracks occurred away from the center as the applied load increased and one of the flexural cracks near the supports developed into a diagonal crack. The first shear crack in each beam was denoted by (**) as shown in Table 13. The addition of 5D, 3D, HY, and SY fibers delayed the first crack by 109%, 98%, 69%, and 1%, respectively. This improvement could be attributed to the high-energy absorption of the fibers, which led to a delay in initial cracks. However, the limited contribution of the synthetic fibers could be explained by the low fiber stiffness and lack of anchorage with concrete. In general, the addition of fibers affected the crack initiation, crack spacing and crack width. The flexure experimental results, which represent average material/beam properties, support these observations as a clear improvement in peak strength and residual strength and were observed as shown in Table 9. The direct measurements of individual cracks using DIC also point to delayed cracking and improved post-cracking response with the addition of fibers. The observed trends, whether through averaged beam response or individual and direct observation of cracks, are consistent with steel fibers offering superior response compared to synthetic fibers. However, it is important to note that the fiber type also influenced the overall behavior. The control beams, SY beams, HY beams, and one 3D beam failed in shear. A shear crack with about 45° angle was the failure mode of the control beams. However, the shear crack for the beams with fibers was controlled due to the fiber distribution, which demonstrated the improved pullout capacity and the ability of the fibers to bridge cracking. Therefore, improved aggregate interlock due to enhancement of the ITZ, anchorage effect of the fibers, and dowel action of the longitudinal bars contributed to the concrete shear resistance. The other three beams failed in flexure (concrete crushing at the compression zone). This showed that the addition of steel fibers improved the splitting tensile strength, while the beam carrying capacity and contributed to higher flexural strength and post cracking behavior.

**Figure 16:**
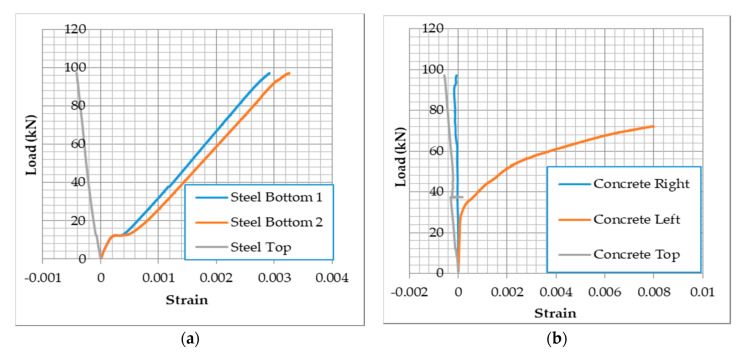
Strain in steel and concrete—RCA-SY-1, (**a**) Strain in bottom and top steel bars, (**b**) Strain in concrete.

Strain in the steel bars and concrete surface were reordered during loading. Figure 16 shows a sample of the load-strain curves for beam-1 with synthetic fibers. From Figure 16a, both bottom bars reached strain ≥ εy (0.00207) around 60 kN. This indicates that the fiber addition improved the flexural strength, which in turn improved the shear capacity. In addition, at the same load, Figure 16b, concrete started to crack, and the strain is about 0.0038 which an indication of improved concrete tensile capacity. It is important to note that location of concrete strain gauges was selected based on estimated shear crack at 45° at 200 mm from the supports, Figure 5. Therefore, it is expected that the strain readings from left and right strain gauges would be different.

### 4.4. Summary of Fiber-Reinforced Experimental Results Versus Proposed Equations

**Figure 17:**
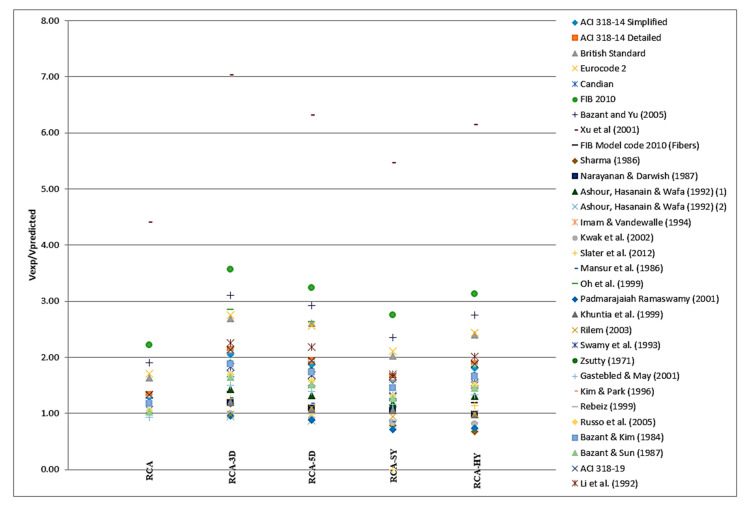
Summary of the Experimental Results versus Proposed Equations.

Results of the shear strength from the experimental investigation for the five concrete mixes presented in the current study are compared to some of the code equations and predication models available in the literature [35,41,42,43,44,45,46,69,71,88,89,90,91,92,93,94,95,96,97,98,99,100,101,102,103,104,105,106,107], as shown in Figure 17. The code and predication equations are divided to equations applicable for (1) mixes with normal weight aggregate and were verified for mixes with recycled aggregate, (2) normal weight concrete only, and (3) fiber reinforced concrete with normal weight aggregate only. In addition, equations are identified if applicable for a specific fiber type. To the authors’ knowledge, there are no specific equations for fiber reinforced concrete with 100% recycled coarse aggregates.

For all experiments, if the ratio between the experimental results and the predicted shear strength is in the range “1” (perfect prediction of shear strength) to “2” (experimental is double the predicted shear strength), then the models used are considered acceptable to calculate *v_c_* for concrete with 100% recycled aggregate. As mentioned previously in Table 1, existing models and codes conservatively predict the effect of RCA and fiber reinforced concrete on shear strength.

#### 4.4.1. Applicability of NW Models Tested on Previous RAC Studies

Refs. [88,100,105] applied NW models on their RAC studies in order to examine their validity. Shear ratios for all studies when applied to the current RAC work range from 1.2–2, with [100,105] the closest prediction to the experimental shear strength value. The ratio discrepancies could be due to several factors such as strength of RA and the amount of mortar remaining on aggregates, affecting the adhesion performance in the ITZ.

#### 4.4.2. Applicability of NW Models on Current RAC Study

Studies that focus on NWA, however, these models were utilized in the current investigation to verify applicability to the RCA, generally conservatively predict the shear strength of RCA [101,102,103,104,106]. The shear ratios range from 0.93–1.3, with the closest to 1.0 being [106] at a ratio of 1.03 and [101] at 0.93. When compared to the previous section that evaluated NW models in previous RAC studies, the range for the shear ratio is smaller and have no anomalies.

#### 4.4.3. Applicability of Fiber Reinforced Concrete with NW Models on Fiber RAC

Studies that focus on steel fibers with NWA and that have been applied to the four different fibers in this study generally conservatively predict the shear strength, with ratios ranging from 0.9–2.8 [46,69,71,92,93,95,97,99,107]. The most accurate prediction for this study is [46], with ratios ranging from 0.94–1.10 for synthetic and 5D fibers respectively. In general, the 3D steel fiber has the highest shear ratio and hybrid fiber has the lowest shear ratio.

#### 4.4.4. Applicability of Code Equations on RAC and Fiber RAC

The codes equations [35,41,42,43,44,45] considered in Figure 17 focus on NWA structural concrete with ratios close to 1, except for the *fib* Model Code 2010 [41] that includes fiber reinforced concrete and is not applied to NWA or RAC. When these codes are applied to RAC, shear strength ratios range from 1.07–2.2 [35,41,42,43,44,45]. The range of the shear strength ratio increases when the same codes are applied to fiber RAC, from 1.15–3.6 [35,41,42,43,44,45]. The *fib* 2010 [41] and ACI 318-19 [35] have been applied to RAC in previous studies, while the other codes mentioned have been specifically for NWA concrete. ACI 318-19 [35] is acceptable for this RCA study, at a conservative ratio of 1.33.

#### 4.4.5. Specific Cases

References [91,98] are the only shear strength study that overestimates the shear strength across the different fiber reinforced concrete, while [88,92,94], also shear strength studies, fluctuate between overestimation and ratios close to 1.0 for the different fiber reinforced concrete [89]. An NWA study that has also been tested with RCA, is the consistent outlier in all experiments, with the lowest shear ratio is RCA and the highest shear ratio being RCA with 3D steel fibers. When considering existing codes, the fib 2010 model code [41] has the largest deviation, with the lowest shear ratio being RCA and the highest shear ratio being RCA with 3D steel fibers. Refs. [46,69,71] have the most consistent shear prediction for all experiments, ranging from 0.9–1.2. The most accurate code is the fib Model 2010 with fiber code, with shear ratios ranging from 1.15–1.46.

In general, RCA without fiber and RCA with synthetic fiber have the largest number of models and codes with shear ratios ranging from 0.8–1.5. However, 3D and hybrid fiber have the lowest number of models and codes with shear ratios in that range, as most of these models are not applicable for RCA with these types of fibers. These statistics correspond with the available literature on the different fibers and their application to concrete with NWA and RCA. In addition, there are limited studies on 3D fiber and applicable models to accurately predict the shear strength of fiber reinforced concrete. 

## 5. Conclusions

The investigation presented in this paper focuses on the evaluation of shear strength of fiber reinforced recycled aggregate and the influence of fiber type/configuration on the concrete shear strength. The experimental program consists of 10 beams utilizing 100% coarse recycled aggregate. The beams were divided into 5 groups (2 beams/group), group1, control specimens, was cast with coarse recycled aggregates without fibers. The other four groups were prepared using 0.75% volumetric ratio of steel fibers 3D and 5D, polypropylene (synthetic) fiber, and hybrid fiber (mix of steel fiber 5D and synthetic fiber). In addition, compressive, splitting tensile and flexural strengths were evaluated for all mixes. The experimental results for shear capacity were compared against predicted values from different design codes and proposed equations in literature. The results from the current study are based on one volumetric ration of fiber and longitudinal reinforcement (ρ); however, the following could be concluded from the findings:

1. Performance of concrete mixes with 100% recycled coarse aggregate is improved by adopting an enhanced mixing procedure and the addition of silica fume. Both helped increase the compressive strength, the cement paste-aggregate bond, enhanced the microstructure and improved the ITZ.
2. The fiber addition improved the crack initiation, propagation and post cracking behavior, which led to ductile behavior and different mode of failures. All fiber types improved the splitting (72 to 140%, 3D, SY, 5D, and HY) and the flexural strengths (8 to 72%, HY, SY, 3D, and 5D); however, the percentage improvement was influenced by the fiber type and configuration. It is important to note that not all fiber types improved the compressive strength.
3. Concrete contribution to shear capacity of the control beams could be predicated by the current codes and shear failure is similar to that of found in the literature for beams without shear reinforcement.
4. The fiber addition led to delay of the first crack, controlled crack width and crack propagation. The first shear crack of all fiber reinforced specimens was initiated when the longitudinal reinforcement reached or closer to yield. All fiber types improved the concrete contribution to the shear capacity. Steel fibers 3D and 5D showed the best performance and increase in shear capacity. The percentage increase was 64.48%, 59.23%, 47%, and 23.4% for the 3D, 5D, HY, and SY, respectively, compared to the control specimens.
5. The improved configuration of the 5D steel fibers increased the anchorage with surrounding concrete, which enhanced the flexural strength and contributed to improving the concrete shear resistance.
6. Synthetic fibers, in this study, showed limited contribution, which could be attributed to low fiber stiffness, lack of anchorage and less pullout capacity compared to the steel fibers. On the other hand, the hybrid mix, RCA-HY, showed relatively mixed results due to the combination of 50% of synthetic fibers and 50% of 5D steel fibers.
7. For RAC, the Canadian and ACI 318 codes could be used to calculate the concrete shear capacity with an acceptable factor of safety.
8. For fiber RAC, the *fib* Model Code 2010 (with fibers) provides an acceptable model to calculate the concrete shear capacity for fiber reinforced concrete.
9. Direct observation of cracking response using DIC, initiation and propagation, enables quantitative assessment of role of fiber addition. The introduction of synthetic fibers delayed shear crack initiation compared to RCA beams with no fiber reinforcement. 3D steel fibers offered additional improvement. In both cases, smaller crack widths were confirmed for fiber reinforced beams compared to RCA at similar loads.
10. Long-term monitoring and evaluation of fiber reinforced recycled aggregate concrete is recommended to validate the finding of the current study.

## Figures and Tables

**Table 1:** Shear capacity of recycled concrete aggregate (RCA) with/without fiber [15,20,21,22,47,48,49,50,51,52,53,54,55,56,57,58,59,60,61,62,63,64,65,66].

Reference	RCA%	Parameters Included in the Evaluation	Remarks
Beams without shear reinforcement			
Fathifazl et al. (2009) [47]	0, 63.5, 74.3	Shear span to depth ratio, beam size, and mix proportioning	Using the proposed proportioning method, there is no major differences between the failure modes, cracking patterns and shear performance of recycled aggregate and conventional beams.
Choi et al. (2010) [22]	0, 30, 50, 100	Beams (Shear)	Shear strength of beams with RA was lower than that of beams with NWA. When RA replacement is less than 50%. Models conservatively predict shear strengths or are close to experimental values.
Yun et al. (2011) [49]	0, 30, 60, 100	Beams (Shear)	Different RCA replacement percentage had minor impact on the deflection and shear strength. Shear failure was sudden and explosive. ACI equations are conservative and valid for RCA shear design.
Arezoumandi et al. (2016) [15]	0, 50, 100	Beams (Shear)	Beams with 100% RA had lower shear strength than those with 50% and 0%. 50% RA beams and 0% beams had similar shear resistance.
Ceia et al. (2016) [50]	0, 20, 50, 100	Prisms and cylinders (Slant Shear test)	Shear strength decreases in specimens with RA. Codes to predict shear strength in specimens produce conservative results.
Katkhuda et al. (2016) [51]	0, 50, 100	Beams (Shear)	Beams with treated RCA have higher shear capacity than beams with untreated RCA. Shear span-to-depth ratios illustrate that treated RCA slightly increases the shear capacity of beams. International codes consider the shear strength of treated RCA beams more conservatively.
Sadati et al. (2016) [52]	0, 50	Beams (Shear)	Shear strength of beams with RA was lower than that of beams without, however beams with a 1:1 ratio of fly ash and RA had lower shear strength.
Waseem et al. (2016) [53]	0, 50, 100	Cylindrical Push-off specimens (Shear)	Normalized shear strength was found to increase when RA replaced NRA in both normal and high-strength concrete. Equations in the PCI code were the most accurate amongst the models reviewed.
Ignjatović et al. (2017) [54]	0, 50, 100	Beams (Shear)	Beams with various levels of RCA but the same amount of shear reinforcement had a ~5% difference in shear strength. Existing codes conservatively predict shear strength of beams with 50% and 100% of RCA and with or without shear reinforcement.
Rahal (2017) [55]	0, 20, 50, 100	Cylindrical Push-off specimens (Shear)	Push-off specimens with RA had a reduction in shear strength. A specimen with 100% RA had a ~29% reduction in shear strength, while a 50% replacement had a ~7% reduction in shear strength.
Wardeh et al. (2018) [58]	0, 100	Beams (Shear)	Shear strength of beams with RA was lower than beams without, regardless of shear span-to-depth ratio. Shear strength results were conservative when compared to existing models.
Al-Jasimee and Abo Dhaheer. (2019) [59]	0, 100	Beams (Shear)	Shear strength of beams with treated RA was higher than beams with untreated RA. Compared to codes, the shear strength of beams with treated RA were more conservatively calculated than beams with untreated RA.
Mohammed et al. (2019) [60]	0, 100	Beams (Shear)	Shear capacity of reinforced beams with RA was similar to reinforced beams with NWA. Shear capacity results were conservative when compared to existing models.
Wardeh et al. (2019) [61]	0, 100	Beams (Shear)	Shear strength of beams with RA was lower than beams without, regardless of the shear span-to-depth ratio. A proposed nonlinear hinge model with the appropriate parameters can be used to predict shear strength of beams with RA.
González-Fontebo and Martinez-Abella (2007, 2009) [20,21]	0, 50	Beams (Shear)	-No significant changes were observed in deflection and ultimate load. Bond failure observed in RA beams was controlled when silica fume was added to the mix.
With and without shear reinforcement			
Fathifazl et al. (2011) [48]	0, 63.5, 74.3	Beams (Shear)	Using the proposed proportioning method, reinforced beams with RA had higher shear strength than conventional beams with RA. When compared to existing models, shear strength results were conservative, provided that beams had a total height less than 450 mm.
Rahal and Alrefaei (2017) [56]	0, 5, 10, 16, 20, 23, 35, 50, 75, 100	Beams (Shear)	Beams with 100% RA had an average of 15% reduction of shear strength. Beams that replaced a smaller grade of coarse aggregate with RA did not have a reduction of shear strength. This finding is only valid with an RA replacement of up to 16%. The normalized shear strength using the square root of the compressive strength, a 20% reduction should be used for beams with RA in order to conservatively predict shear strength.
Pradhan et al. (2018) [57]	0, 100	Beams (Shear)	Beams with RA and the same reinforcement as NWA beams had less shear strength, indicating less shear resistance provided by the concrete. Existing equations are unable to predict shear strength for beams with RA and shear reinforcement.
Li et al. (2020) [62]	30, 40, 50, 60	Beams (Shear)	As the shear-span to depth ratio increases in beams, the shear capacity decreases. Shear strength results were conservative when compared to existing models.
Al Mahmoud et al. (2020) [63]	0, 30, 100	Beams (Shear)	Shear strength of beams decreased as the RA replacement ratio increased. Shear strength results were conservative when compared to existing models. Beams with RA had more conservative results than beams without.
RCA with fibers			
Etman et al. (2018) [64]	0, 15, 30, 45	Beams (Shear)	Beams with a higher RA replacement ratio had a higher decrease in shear strength. Adding internal short fibers along with RA, not only compensates for the decrease in shear strength but also led to increase in the shear strength.
Chaboki et al. (2019) [65]	0, 50, 100	Beams (Shear)	Beams without transverse reinforcement increased in shear strength when steel fibers were added. Increasing the RA replacement ratio improved the shear behavior with different transverse reinforcement spacing.
Sayhood et al. (2019) [66]	0, 100	Beams, Cylinders, Cubes, Prims (Shear)	Shear strength of beams with RA was lower than beams without. Shear strength of beams with steel fiber was higher than beams without.

**Table 2:** Equations for *v_c_* from common code of practice [35,41,42,43,44,45,46].

Reference	Concrete Shear Strength
ACI 318-14 (simplified) [45]	$V_{c}=\left(\frac{\lambda \sqrt{{f^{\prime }}_{c}}}{6}\right)b_{w}d$
ACI 318M-14 (detailed) [45]	$V_{c}=\left(0.16\sqrt{{f^{\prime }}_{c}}+17\frac{\rho_{l}}{\frac{a}{d}}\right)\leq 0.29\sqrt{{f^{\prime }}_{c}}$
BS 8110 (British code) [44]	$Vc=\frac{0.79}{\gamma_{m}}{\left(\frac{100As}{bd}\right)}^{\frac{1}{3}}{\left(\frac{400}{d}\right)}^{\frac{1}{4}}$
Eurocode 2 [43]	$V_{Rd,c}=\frac{0.8}{\gamma_{c}}{\left(100f_{ck}\right)}^{\frac{1}{3}}\left(1+\sqrt{\frac{200}{d}}\right)bd$
Canadian code [42]	$V_{c}=0.2\sqrt{{f^{\prime }}_{c}}b_{w}d$
*fib* 2010 [41]	$V_{Rd,c}=k_{v}\frac{\sqrt{f_{ck}}}{\gamma_{c}}bz$
*fib* Model Code 2010 (fibers) [41]	$V_{R}=\frac{0.18kbd_{e}}{\gamma_{c}}{\left(100\rho_{1}f_{cm}\left(1+7.5\frac{f_{Ftu}}{f_{ct}}\right)\right)}^{1/3}$
RILEM 2004 [46]	$V_{frc}=0.18\left(1+\sqrt{\frac{200}{d}}\right){\left(100*\frac{A_{f}}{b_{w}d}*\left(1+7.5\frac{f_{Ftuk}}{f_{ctk}}\right)*f_{ck}\right)}^{1/3}b_{w}d$ for Steel Fibers $V_{c}=0.15*\sqrt[{3}]{3\left(\frac{d}{a}\right)}*k{\left(100*\rho *{f^{\prime }}_{c}\right)}^{\frac{1}{3}}$ $V_{SYF}=\frac{1600-d}{1000}*0.5\frac{d}{a}f_{e,3}$ $V_{frc}=V_{c}+V_{SYF}$ for Synthetic Fibers
ACI 318-19 [35]	$V_{c}=\left(0.17\lambda \sqrt{{f^{\prime }}_{c}}\right)b_{w}d$ For A_v_ ≥ A_v,min_ $Vc=0.66\lambda_{s}\lambda {\left(\rho_{w}\right)}^{1/3}\sqrt{{f^{\prime }}_{c}}+\frac{N_{u}}{6A_{g}}$ For A_v_ < A_v,min_

*V_c_* concrete contribution to shear strength, ${f^{\prime }}_{c}$ concrete compressive strength in MPa, d distance from extreme compression fiber to centroid of longitudinal tension reinforcement, mm, *b_w_* web width, mm, *ρ_w_* ratio of As to *b_w_d*, As area of nonprestressed longitudinal tension reinforcement, mm^2^, A_v_ area of shear reinforcement within spacing s, mm^2^, A_v,min_ minimum area of shear reinforcement within spacing s, mm^2^.

**Table 3:** Summary of recycled aggregate properties.

Test Name		Code	Sample Size (Grams)	Testing Events	Number of Samples	Test Results
Absorption Test		ASTM C642—13 [74]	500	24 h	6	6.07~7.51
				48 h	6	5.77~6.07
				72 h	6	4.60~5.47
Relative Density	Specific Gravity (Oven Dry)	ASTM C127-15 [75]	2000~2700	-	6	2.31~2.35
	Specific Gravity (SSD)			-	6	2.46~2.49
	Apparent Specific Gravity			-	6	2.72~2.74
LA Abrasion		ASTM C131/C131M-20 [76]	Grade B% (4580 ± 25)		3	% (Weight loss) Grade B (% 35)
			Grade C% (3330 ± 20)			Grade C (%31)

**Table 4:** Summary of fibers properties.

Property	Strux(90/40) Synthetic [79]	3D Steel Fiber [78]	5D Steel Fiber [78]
Specific gravity	0.92	7.8	7.8
Modulus of elasticity (GPa)	9.5	210	210
Tensile strength (MPa)	620	1345	2300
Melting point	160 °C	NA	NA
Ignition point	590 °C	NA	NA
Length (mm)	40	35	60
Diameter (mm)	0.44	0.55	0.9
Codes	ASTM C1116	ASTM C1609/C160M-05	
	ANSI/SDI C-1.0		

**Table 5:** Mix proportioning.

	No Fiber	With Fiber
Type I cement	0.12	0.12
Silica Fume	0.05	0.05
Water	0.19	0.19
Recycled coarse aggregate	0.37	0.37
Normal weight fine sand	0.27	0.2625
Fiber	0	0.0075
Total Volume	1	1

**Table 6:** Summary of the experimental investigation—mechanical properties.

Test Name	Test Specifications	Sample Size (mm)	Testing Events (Days)	No. of Specimens Per Test
Compressive Strength—Cube	BS 1881-116:1983 [79]	150 × 150 × 150	28 and 90	2
Splitting tensile Strength—Cylinder	ASTM C496/C496M—17 [80]	200 × 100	28 and 90	2
Flexural strength—Prism	ASTM C1609/C1609M—19 [81]	100 × 100 × 500	28 and 90	2

**Table 7:** Summary of the 90-day mechanical properties results.

	Mechanical Property	Compressive Strength		Splitting Tensile Strength	
Mix ID		Avg. *f′c* (MPa)	% Difference	Splitting Tensile strength (MPa)	% Difference
RCA (Control)		64.50	-	2.77	-
Fiber reinforced RCA					
RCA-3D		68.00	5.43	4.78	72.5
RCA-5D		75.67	17.31	6.19	123.47
RCA-SY		64.00	−0.78	5.36	93.5
RCA-HY		70.50	9.30	6.67	140.8

**Table 8:** Failure modes of cube and cylinder specimens from all mixes.

Mix	Cube Compressive Strength	Split Tension
RA	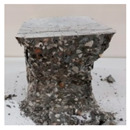 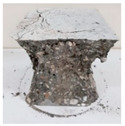	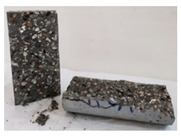 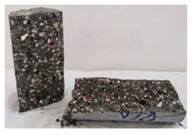
RCA-3D	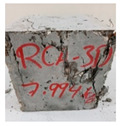 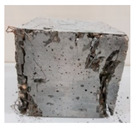	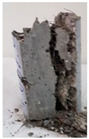 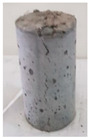
RCA-5D	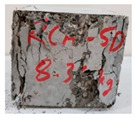 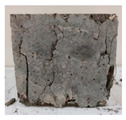	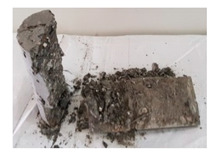 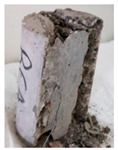
RCA-SY	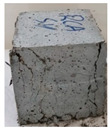 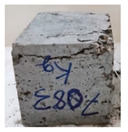	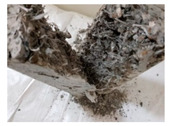 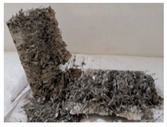
RCA-HY	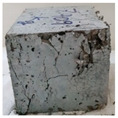 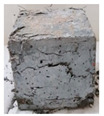	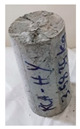 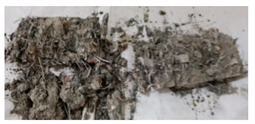

**Table 9:** Summary of the flexural strength results.

Mix ID	First-Peak Strength (MPa)	% Difference *	Peak Strength (MPa)	% Difference *	Residual Strength (L/600) (MPa)	Residual Strength (L/150) (MPa)
RCA (Control)	0.42	-	6.25	-		
Fiber reinforced RCA						
RCA-3D	0.43	2.38	7.73	23.68	6.27	4.50
RCA-5D	0.43	2.38	10.89	74.24	9.28	5.40
RCA-SY	0.80	90.48	6.94	11.04	5.34	3.65
RCA-HY	0.46	9.52	6.77	8.32	4.82	3.49

* Compared to RCA without fiber.

**Table 10:** Summary of the results for all beams.

Beam ID	$\mathrm{Compressive}\;\mathrm{Strength}\;({f^{\prime }}_{c})$ (MPa)	Ultimate Load (kN)	Failure Load (kN)	Shear Load (*V_c_*) (kN)	α *	α`	Shear Load *** % Difference	$\mathrm{Shear}\;\mathrm{Resistance}/\sqrt{{f^{\prime }}_{c}}$	Failure Mode	Δy (mm)	Δmax (mm)	Ductility Index Δmax/Δy
RCA 1		82.8	66.06	41.4					Shear	6.43	9.58	
RCA 2		80.6	64.72	40.3					Shear	7.73	8.85	
Average	64.50	81.7	65.39	40.85	0.21	0.88	-	0.0051		7.08	9.22	1.3
RCA-3D-1		128.7	120.47	64.35					Flexure	6.42	14.58	
RCA-3D-2		140.06	136.28	70.03					Shear- Flexure	6.51	14.52	
Average	68.00	134.38	128.38	67.19	0.34 **	1.41 **	64.48	0.0081		6.47	14.55	2.25
RCA-5D-1		130.16	121.96	65.08					Flexure	4.42	14.66	
RCA-5D-2		130.02	121.05	65.01					Flexure	5.50	14.2	
Average	77.00	130.09	121.51	65.05	0.31 **	1.28 **	59.23	0.0074		4.96	14.43	2.91
RCA-SY-1		97.22	96.59	48.61					Shear	5.49	10.61	
RCA-SY-2		104.48	104.48	52.24					Shear	8.03	10.58	
Average	64.00	100.85	100.54	50.43	0.26 **	1.09 **	23.44	0.0063		6.76	10.6	1.57
NWA-HY-1		123.8	123.8	61.9					Shear	5.36	19.93	
NWA-HY-2		116.4	116.4	58.2					Shear	7.80	12.64	
Average	70.50	120.1	120.1	60.05	0.3 **	1.24 **	47.00	0.0072		6.58	11.79	1.79

$\alpha =\frac{v_{c}}{b_{w}d\sqrt{{f^{\prime }}_{c}}},$ * $\alpha =\frac{v_{c}}{b_{w}d\sqrt{{f^{\prime }}_{c}}}$, for A_v_ ≥ A_v,min_ [35], $\alpha^{\prime }=\frac{v_{c}}{{\left(\rho_{w}\right)}^{1/3}\sqrt{{f^{\prime }}_{c}}b_{w}d}$ for A_v_ < A_v,min_, [35] ** includes the effect of fiber *** compared to shear resistance of RCA beams.

**Table 11:** Summary of mechanical properties of fiber reinforced recycled concrete from literature compared to the current study [27,29,30,32,34,40,66,86,87]

Authors	RCA%	Fibers%	Fiber	Parameters Tested			Testing Dates (Days)	Remarks
				Compressive Strength	Tensile Strength	Flexural Strength		
Ahmadi et al. (2017) [40]	0%, 50%, 100% *	0%, 1% *	Recycled Steel Fibers	−11.94%	48.28%	22.86%	28	Fibers reduced compressive strength however improved Mechanical properties
Afroughsabet et al. (2017) [29]	0%, 50%, 100% *	0%, 1% *	Steel Fibers	7.24%	52.93%	79.42%	Compressive strength at 91 remaining tests at 28	Fibers Improved Mechanical properties	
Gao et al. (2017) [34] Gao et al. (2018) [30]	0%, 100% *, 0%, 30%, 50%, 100% *	0%, 1% *, 0%, 0.5%, 1% *, 1.5%, 2%	Steel Fibers	4.00%	-	12.40%	28	Fibers has inconsiderable effect
Ahmed et.al (2020) [32]	0%, 50%, 100% *	0%, 0.15%, 0.3%, 0.45%, 0.6%, 0.75% *, 0.9%	Synthetic Fibers	−8.13%	−19.45%	−11.80%	28	Fibers reduced mechanical properties of RCA
Chen et al. (2014) [27]	0%, 100% *	0%, 0.5%, 1% *, 1.5%	Steel Fibers	−12.12%	-	-	28	Reduced compressive strength of RCA
Ramesh et al. (2018) [86]	0%, 30%, 50%, 70%, 100% *	0%, 0.3%, 0.5%, 0.7% *, 1.0%	Steel Fibers	24.70%	93.10%	-	28	Fibers Improved Mechanical properties
Kazmi et al. (2019) [87]	0%, 50%, 100% *	0%, 0.5%, 1% *	Synthetic Fibers	9.00%	-	-	28	Fibers improved the compressive strength and stress-strain curves
Sayhood et al. (2019) [66]	0%, 100% *	0%, 0.5%, 1% *, 1.5%	Steel Fibers	27.42%	33.33%	32.76%	NA	Fibers improves the mechanical properties
Chaboki et al. (2019) [60]	0%, 50%, 100% *	0%, 1% *, 2%	Steel Fibers	−1.11%	1.49%	-	28	Fibers improved shear behavior and inconsiderable effect on mechanical properties
Current Study	100%	0.75%	3D steel fibers	5.43%	72.83%	23.65%	90	Fibers Improved Mechanical properties
			5D steel fibers	17.31%	123.55%	74.12%		
			Synthetic Fibers	−0.78%	93.62%	10.99%		
			Hybrid (5D + SY)	9.30%	141.16%	8.30%		

* Results considered in the comparison.

**Table 12:** Crack distrubtion—beams after testing.

	Sample #	Sample 1	Sample 2
Mixes			
RCA		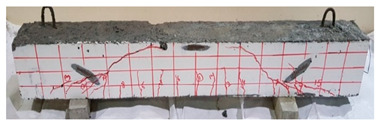	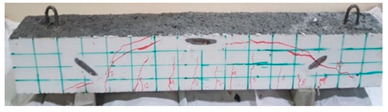
RCA-3D		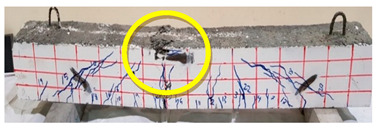	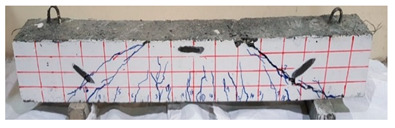
RCA-5D		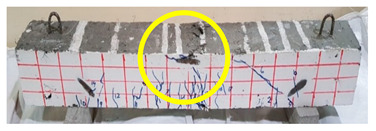	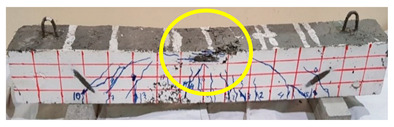
RCA-SY		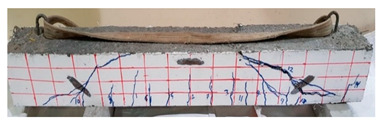	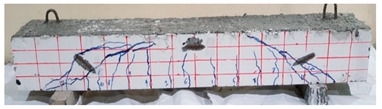
RCA-HY		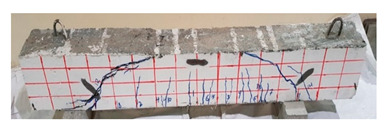	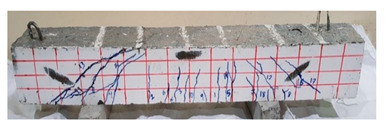

**Table 13:** Summary of crack, corresponding loads, and strain.

Beam	RCA 1	RCA 2	RCA-3D-1	RCA-3D-2	RCA-5D-1	RCA-5D-2	RCA-SY-1	RCA-SY-2	RCA-HY-1	RCA-HY-2	
**Crack #**	**Load (kN)**									
**1**	20	18.71	43.09	33.44	40.8	40.15	18.06	21.2	37.18	28.09
**S_strain**	0.0006	0.0005	0.0009	0.0006	0.0013	0.001	0.0001	0.0002	0.0009	0.0005
**C_Strain**	0.00005	0.00002	0.0001	0.0001	0.0001	-	0.0001	-	-	-
**2**	21.05	19.11	44.9	34.65	63.59	41.42	18.88	24.5	39.05	29.02
**3**	21.74	19.73	45.78	37.11	66.13	45.83	19.44	26.07	40	30.31
**4**	23.25	21.57	46.51	42.28	79.57	51.1	19.89	29.29	41.44	32.28
**S_strain**	0.0007	0.0006	0.001	0.0008	0.0026 *	0.0012	0.0007	0.005	0.0011	0.0007
**C_Strain**	0.00007	0.00003	0.0002	0.0001	0.0022	-	0.0006	-	-	-
**5**	24.17	22.3	54.47	48.77	80.2 **	52.31	21.45	33.71	42.68	40.4
**6**	25.18	36.07	55.48	56.76	81.38	52.78	27.53	38.49	46.11	44.1
**7**	26.96 **	37.17 **	56.1	60.07	84.81	54.16	31.12	41.3	52.58	46.2
**8**	30.1	39.27	57.66	78.46	90.34	67.66	33.63	44.42	55.47	53.4
**S_strain**	0.0009	0.0011	0.0013	0.0018	0.003	0.0017 *	0.0012	0.001	0.0015	0.0013
**C_Strain**	0.0001	0.00003	0.0005	0.0006	0.002	-	0.001	-	-	-
**9**	45	45.54	65.95 **	79.98	100.2	88.15	33.91	53.6	59.78	68.45
**10**	47	65.11	69	81.24	118.8	91.26 **	44.23 **	63.8	69.01	70.31
**11**	52.48	66.25	69.84	83.47	122.3	97.12	54.05	66.07 **	81.84	71.49
**S_strain**	0.0016	0.0019	0.0017	0.0019*	0.0048	0.0013	0.0018	0.0016 *	0.0022 *	0.0017
**C_Strain**	0.0004	0.0061	0.001	0.0007	0.0029	-	0.0024	-	-	-
**12**	62.1	68.36	75	85.91 **	122.3	98.03	70.32	80.7	83.28**	76.09
**13**	63.63		78.22	101.1	122.9	101.6	88.92	82.4	85.51	81.42 **
**14**	64		79.5	115.4	124	102.4	79.1		100.4	83.56
**15**	68.8		93.7	135.3	124.4	112.4			114.6	88.2
**16**			99.9	135.3	125.8	126.5				97.69
**17**			100.3	136.1	128.3	127.7				101.2
**S_strain**			0.0025 *	0.011	0.015	0.011				0.0026 *
**C_Strain**			0.0034	-	0.0031					
**18**			101.5							106.9
**19**			105.3							115.7
**20**			119.95							
**21**			121.6							
**22**			121.9							

S_strain = strain in bottom bar, C_strain = strain in concrete, * Bottom- started to yield/yielding (ε ≥ 0.00207) ** Shear crack.
